# High g-Force Rollercoaster Rides Induce Sinus Tachycardia but No Cardiac Arrhythmias in Healthy Children

**DOI:** 10.1007/s00246-016-1477-5

**Published:** 2016-09-21

**Authors:** Guido E. Pieles, Victoria Husk, Teresa Blackwell, Deirdre Wilson, Simon M. Collin, Craig A. Williams, A. Graham Stuart

**Affiliations:** 1 Bristol Congenital Heart Centre, The Bristol Heart Institute, University Hospitals Bristol NHS Foundation Trust, Bristol, UK; 2 National Institute for Health Research (NIHR) Cardiovascular Biomedical Research Unit, Bristol Heart Institute, University of Bristol, Bristol, UK; 3 Centre for Child and Adolescent Health, School of Social and Community Medicine, University of Bristol, Bristol, UK; 4 Children’s Health and Exercise Research Centre, College of Life and Environmental Sciences, University of Exeter, Exeter, UK

**Keywords:** Cardiology, Arrhythmia, Rollercoaster, g-force

## Abstract

Theme park operators and medical professionals advise children with heart conditions against using rollercoaster rides, but these recommendations are not evidence-based. The underlying assumption is that the combination of adrenergic stimulation through stress and acceleration might trigger arrhythmias in susceptible individuals. We conducted a cross-sectional observational study to assess heart rate and rhythm in healthy children during commercial rollercoaster rides. Twenty healthy children (9 male) aged 11–15 (mean 13.3 ± 1.4) years underwent continuous heart rate and rhythm monitoring (2-lead ECG) from 5 min before until 10 min after each of 4 high speed (>50 km h^−1^), high g-force (>4) commercial rollercoaster rides. Total recording time was 13 h 20 min. No arrhythmic events were detected. Resting heart rate was 81 ± 10 b min^−1^ and increased to 158 ± 20 b·min^−1^ during rides. The highest mean HR (165 ± 23 b min^−1^) was observed on the ride with the lowest g-force (4.5 g), but one of the highest speeds (100 km h^−1^). Anticipatory tachycardia (126 ± 15 b min^−1^) within 5 min was frequently observed. A 10 min recovery HR (124 ± 17 b min^−1^) was 56 % greater than resting HR. The speed and g-force experienced on roller coasters induce sinus tachycardia but do not elicit pathological arrhythmias in healthy children.

## Introduction

Theme parks are popular with children and adults. In the UK, the three biggest theme parks had 6.9 million visitors in 2014, a 4.7 % increase compared to 2013 [1]. Attendance at a theme park is regarded as safe, with an injury risk requiring hospitalisation of 1 in 24 million [2]. In the US, approximately 40 deaths over 10 years were attributable to rollercoaster rides, five of which were in children with a suspected cardiac or respiratory cause [3]. Although theme park ride operators and medical professionals advise children with heart conditions against using the rides, these recommendations are based on consensus, not on scientific evidence.

A primary concern is that the combination of adrenergic stimulation through stress and acceleration (g-force; g) experienced on roller coasters might trigger arrhythmias in susceptible individuals [4]. Studies from aviation science have demonstrated that high g can provoke atrial and ventricular ectopics, paroxysmal supraventricular tachycardia, paroxysmal atrial fibrillation but also sustained ventricular tachycardias [5]. Research on the cardiovascular effect of rollercoaster rides has been confined to adults participating in older style rides which have relatively low g generation. However, these studies have reported asymptomatic supraventricular arrhythmias, non-sustained ventricular tachycardia and even aortic dissection [6, 7]. To the best of the authors knowledge, there have been no reported studies on the cardiovascular effects of rollercoaster rides in children or adolescents. This is the first study to investigate the effect of modern high g rollercoaster rides on heart rhythm in healthy children.

## Patients and Methods

Twenty healthy children (9 male), mean age of 13.3 ± 1.5 years underwent a cardiovascular examination, medical and family history questionnaire and 12-lead ECG prior to the study. Patients with an abnormal cardiovascular examination, history or ECG were excluded from the study. Whilst at the theme park, participants wore a 2-lead ambulatory ECG monitor (R test evolution 4, Novacor©). After manual activation, data were recorded continuously from 5 min before rollercoaster rides (anticipatory period) until 10 min after the ride (recovery period) on four rides. The maximum g was 5 g (mean 4.7 ± 0.2 g), maximum speed was 130 km h^−1^ (mean 92 ± 48.2 km h^−1^), and the average ride time was 82 ± 36 s with breaks of a minimum of 45 min between rides (Table 1). Data were analysed using dedicated offline software (RT Soft Ultima, Novacor©) by one investigator trained in paediatric ECG analysis (GEP), blinded to the type of ride and identification of participant.

**Table 1:** Rollercoaster ride characteristics and heart rate (HR) measurements

Max Speed (km ^−1^)	Max g-force	Max height (m)	Length of ride (m)	Duration of ride (s)	Sex of rider	Number of riders	Baseline (resting) HR (b min^−1^)	Anticipatory HR (b min^−1^)	Max HR (b·min^−1^)	Recovery HR at 10 min (b min^−1^)	HR change from resting to max (b min^−1^)	HR change from resting to recovery (b min^−1^)
*Ride 1*												
100	4.5	39	775	125	Both	17	83 (9)	122 (13)	165 (23)	126 (12)	82 [70, 95]^***^	43 [37, 50]^***^
					Boys	7	84 (6)	114 (10)	152 (21)	123 (10)	93 [78, 107]^***^	46 [36, 90]^***^
					Girls	10	82 (11)	128 (12)	174 (20)	128 (13)	68 [47, 89]^***^	39 [31, 47]^***^
*Ride 2*												
130	4.5	62.5	500	24	Both	17	81 (11)	133 (11)	158 (17)	119 (14)	78 [70, 86]^***^	38 [30, 47]^***^
					Boys	9	81 (9)	126 (7)	152 (15)	113 (9)	71 [61, 81]^***^	33 [22, 43]^***^
					Girls	8	81 (12)	140 (10)	166 (16)	126 (17)	85 [72, 98]^***^	46 [29, 62]^**^
*Ride 3*												
89	4.8	30	700	100	Both	15	82 (10)	128 (19	159 (17)	119 (16)	77 [68, 87]^***^	40 [30, 49]^***^
					Boys	5	82 (7)	124 (12)	151 (10)	115 (23)	69 [54, 83]^***^	33 [7, 59]^*^
					Girls	10	82 (11)	130 (23)	163 (19)	122 (12)	82 [68, 95]^***^	43 [33, 54]^***^
*Ride 4*												
50	5	18	–	120	Both	10	79 (10)	118 (13)	142 (19)	139 (25)	63 [53, 72]^***^	57 [41, 73]^***^
					Boys	4	81 (12)	113 (13)	144 (18)	135 (30)	63 [38, 88]^**^	54 [18, 89]^*^
					Girls	6	78 (9)	128 (3)	140 (21)	143 (23)	62 [48, 76]^***^	60 [33, 88]^**^
*All rides*												
92 (48)	4.7 (0.2)	37 (16)	658 (116)	92 (41)	Both	17	81 (10)	126 (15)	158 (20)	124 (17)	76 [71, 81]^***^	44 [39, 49]^***^
					Boys	9	81 (9)	120 (11)	151 (16)	120 (18)	68 [62, 75]^***^	38 [31, 45]^***^
					Girls	11	81 (11)	132 (15)	163 (21)	128 (16)	82 [75, 89]^***^	48 [41, 55]^***^

HR measurements and characteristics of ‘All rides’ are shown as mean (SD). HR change from resting is shown as mean [95 % CI]. Ride 1 is a ‘wing coaster’, on which the rider experiences five inversions (loops). Ride 2 is a launch rollercoaster, accelerating from 0 to 130 km in under 2 s to a height of 205 ft, before dropping back down. Ride 3 is a ‘Euro-Fighter’ rollercoaster, 100 s of a haunted house theme and blind vertical drops (free falls). Ride 4 has long arms, which spin relentlessly around 360 degree pivots for 180 s

* *p* < 0.05; ** *p* < 0.01; *** *p* < 0.001

### Statistical Analysis

All data are presented as mean (SD) and mean difference [95 % CI] unless otherwise stated. The mean resting, anticipatory, maximum and recovery heart rates (HR) were compared with baseline HR for each ride using Student’s t test. Random effects linear regression models adjusted for age and resting HR were used to investigate differences in maximum, recovery and anticipatory HR by ride and by sex. All analyses were performed using Stata (StataCorp 2013. Stata Statistical Software: Release 13. College Station, TX: StataCorp LP).

## Results

**Fig. 1:**
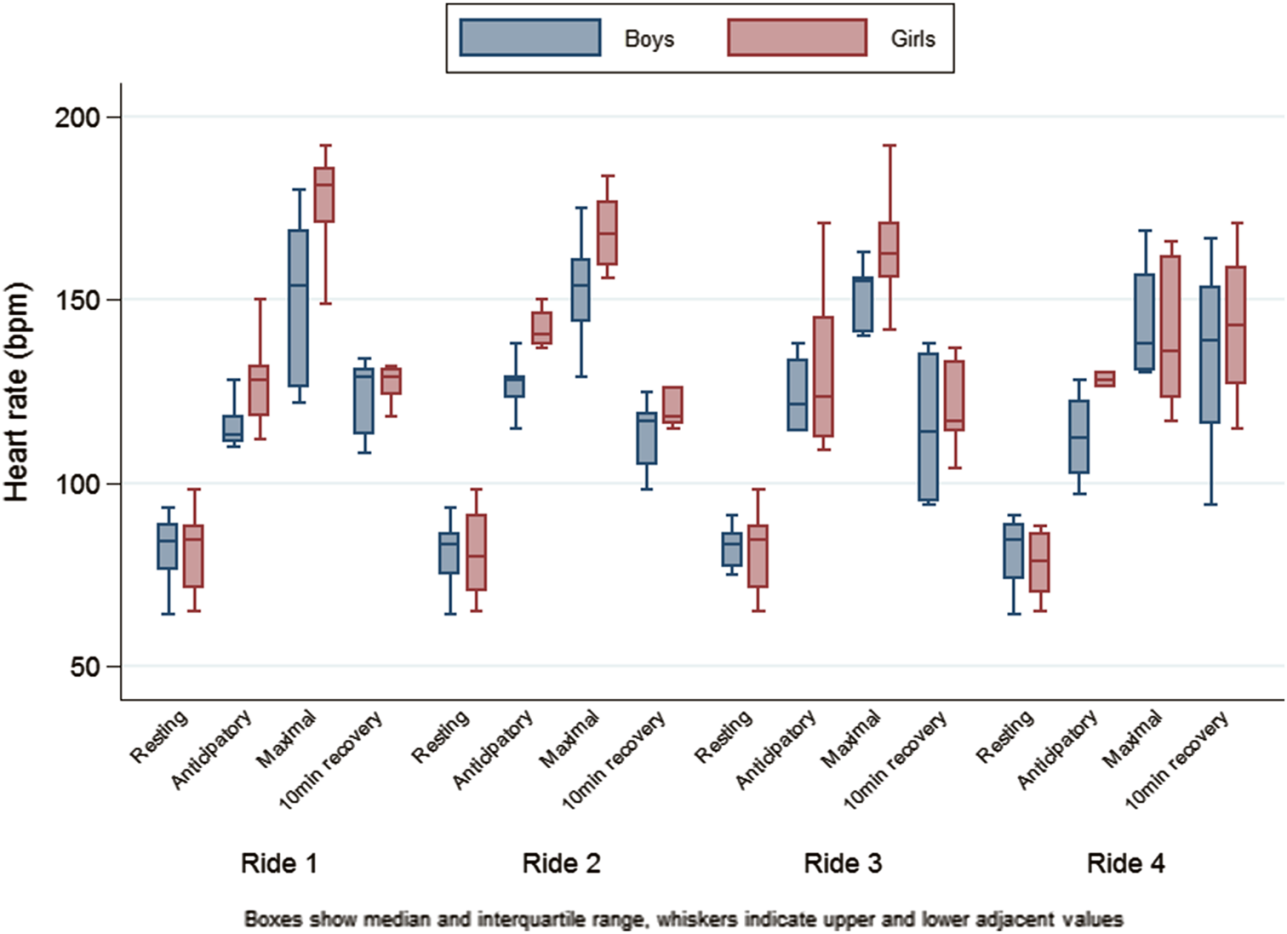
HR measurements (resting, anticipatory, maximal and 10 min recovery) across the four rollercoaster rides

The baseline 12-lead ECG showed a mean resting HR of 81 ± 10 b min^−1^. No baseline 12-lead ECG abnormalities were detected. During the data collection at the theme park, total ECG recording time was 13 h 20 min with 59 individual ride recordings. Nineteen out of twenty participants experienced sinus tachycardia (HR > 100 b min^−1^), with 214 episodes of tachycardia recorded (Table 1; Fig. 1). No pathological arrhythmias were detected.

All rides induced a significant HR increase (*p* < 0.001). Different effect on HR of speed *vs* g was also investigated. Maximum HR on Ride 1, with higher speed but lower g compared to Ride 4, was higher by 20 [95 % CI 9–30] b min^−1^, *p* < 0.001. Ride length did not affect the degree of tachycardia.

Overall, there was a slight tendency for boys to have lower maximum HR than girls (difference −11 [−28 to 7] b min^−1^, *p* = 0.23) (Table 1; Fig. 1).

There was a significant anticipatory tachycardia (mean difference from resting HR 46 [41–51] b min^−1^, *p* < 0.001) in 96 % of data points. Anticipatory HR was lower in boys than in girls (difference −18 [−28 to −8] b min^−1^, *p* = 0.001).

HR at 10 min recovery was significantly higher than resting HR (difference 44 [39–49] b min^−1^, *p* < 0.001). There were no differences in mean HR at 10 min recovery between Rides 1–3, although it was slightly higher for Ride 4 with the highest g compared to Ride 1 (difference 14 [1–26] b min^−1^, *p* = 0.03). Recovery HR was also lower in boys than in girls (difference −12 [−23 to −1] b min^−1^, *p* = 0.03) (Table 1).

## Discussion

The aim of this study was to investigate the cardiovascular stress elicited during rollercoaster rides in young boys and girls. Specifically, we recorded 2-lead ECG traces to establish the incidence of any atrial and ventricular ectopics, paroxysmal supraventricular tachycardia, paroxysmal atrial fibrillation or sustained ventricular tachycardia. Although this is a small study, it is the first report of the effects of high speed and high g on HR response and arrhythmia induction in healthy children participating in a high g roller coaster. This lack of paediatric data is surprising considering the numbers of riders annually in theme parks around the world.

Our results showed a significant increase in HR during rollercoaster rides from baseline resting HR (*p* < 0.001 for all rides), the levels of which are comparable to HR during moderate exercise. Although sudden sympathetic activation, as experienced on roller coasters, is one mechanism of pathological arrhythmia induction, in contrast to adult studies, we have, however, not observed any episodes of abnormal rhythm in children. However, significant sinus tachycardia occurred in 95 % of children which is a much higher frequency than the 44 % reported in adults [7].

Remarkably, a tachycardic response was not confined to periods on rides, but we recorded significant (*p* < 0.001) tachycardia 5 min before the ride indicating that part of the tachycardia effect is not related to speed or g but is an anticipatory response, which was more pronounced in girls.

We also investigated the differential effects of speed and g on HR response. Rollercoaster rides with the highest speeds (rides 1 and 2) evoked a more pronounced tachycardia response than rides with higher g (rides 3 and 4). This suggests that acceleration to a greater maximal speed might play a greater part in triggering tachycardia than the effect of the g exerted. In contrast, a higher g but lower speed had a negative effect on HR recovery (Table 1). Sample size was a limitation.

## Conclusion

This pilot study showed for the first time that there is significant sinus tachycardia, but no arrhythmia induction in response to anticipation, stress, catecholamine release and g exerted by modern theme park rides in healthy children. Permission to participate in rollercoaster rides and similar fairground activities is often sought by families of children with a variety of medical conditions. Although this is a small study, we hope that the detailed data obtained can help the paediatrician to give more informed advice.
